# Microglial Activation Is Associated with Hippocampal Synaptic Degeneration and Cognitive Deficits Following Repeated Propofol Exposure

**DOI:** 10.3390/ijms27146293

**Published:** 2026-07-15

**Authors:** Liyun Deng, Mengchan Su, Ying Cui, Jiahui Wu, Guo Chen, Ruotian Jiang, Chan Chen

**Affiliations:** 1 Department of Anesthesiology, West China Hospital, Sichuan University, Chengdu 610041, China; dengliyundoctor@163.com (L.D.); 18228015989@163.com (Y.C.); wujiahui_scu@foxmail.com (J.W.); chenguohx2023@163.com (G.C.); ruotianjiang@scu.edu.cn (R.J.); 2 Laboratory of Anesthesia and Critical Care Medicine, National-Local Joint Engineering Research Center of Translational Medicine of Anesthesiology, West China Hospital, Sichuan University, Chengdu 610041, China; sumengchan_scu@foxmail.com; 3 Department of Pain Management, West China Hospital, Sichuan University, Chengdu 610041, China

**Keywords:** repeated propofol exposure, conditioned place preference, cognitive impairment, microglial activation, synaptic degeneration, minocycline

## Abstract

Propofol is a widely used intravenous anesthetic with recognized abuse potential; however, the effects of repeated propofol exposure on hippocampal function and cognition remain poorly understood. In the present study, we established a rat model of repeated subanesthetic intraperitoneal propofol administration and demonstrated that propofol induced robust conditioned place preference, indicating rewarding properties. Propofol-exposed rats exhibited significant impairments in hippocampus-dependent cognitive tasks. Transcriptomic analysis revealed marked transcriptional alterations enriched in pathways related to synaptic organization and plasticity following repeated propofol exposure. Consistently, Western blotting, transmission electron microscopy, and Golgi staining demonstrated pronounced reductions in dendritic spine density and synaptic integrity within the hippocampus. Moreover, aberrant microglial activation was observed in the hippocampus and was closely associated with synaptic degeneration and cognitive deficits. Importantly, pharmacological inhibition of microglial activation with minocycline effectively ameliorated propofol-induced cognitive impairment and synaptic degeneration. Collectively, these findings suggest that microglial activation is associated with hippocampal synaptic degeneration and cognitive deficits following repeated propofol exposure and may contribute to these pathological changes.

## 1. Introduction

Propofol is one of the most widely used intravenous anesthetics because of its rapid onset and favorable recovery profile [1,2]. However, increasing clinical and preclinical evidence indicates that propofol possesses substantial abuse liability. Since the first report of propofol dependence in 1992, numerous cases of recreational use, dependence, and fatal overdose have been documented [3,4,5,6,7]. Consistent with these observations, animal studies have demonstrated that repeated propofol exposure induces robust reward-related behaviors, including conditioned place preference (CPP), through activation of neural reward circuits [8,9,10,11,12]. Despite growing concerns regarding propofol abuse, its neurobiological consequences remain poorly understood.

Cognitive impairment is a common consequence of substance use disorders and contributes to poor treatment outcomes and relapse vulnerability [13,14,15]. Repeated exposure to addictive substances induces persistent neuroadaptations within neural circuits governing reward processing and cognition, resulting in impaired synaptic plasticity and memory function [16,17,18]. The hippocampus is particularly vulnerable to drug-induced neuroplastic alterations because of its central role in learning and memory and its close functional interaction with mesolimbic reward pathways. Previous studies have shown that multiple addictive substances impair hippocampal synaptic integrity and cognitive performance [19,20]. Emerging evidence suggests that repeated propofol exposure similarly induces hippocampal dysfunction. Recent studies, including our own work, have demonstrated that repeated propofol exposure promotes hippocampal neuroinflammation, synaptic dysfunction, neuronal senescence, and cognitive decline [21,22]. However, the cellular mechanisms linking these pathological alterations to hippocampal dysfunction remain unclear.

Synaptic remodeling is a fundamental substrate of learning and memory [23,24,25,26,27]. Microglia, the resident immune cells of the central nervous system, are key regulators of synaptic homeostasis [28,29]. Dysregulated microglial activation has been increasingly implicated in synaptic degeneration and cognitive dysfunction across a range of neurological disorders [30,31]. Notably, accumulating evidence indicates that addictive substances can induce microglial activation and maladaptive synaptic remodeling [32,33,34,35]. Given the established involvement of neuroinflammation and synaptic dysfunction following repeated propofol exposure, microglia–synapse interactions may represent a critical mechanism underlying hippocampal pathology. However, whether aberrant microglial activation contributes to hippocampal synaptic degeneration and cognitive impairment following repeated propofol exposure remains unclear.

In the present study, we investigated the impact of repeated subanesthetic propofol exposure on hippocampal synaptic integrity and cognitive function. We hypothesized that repeated propofol exposure induces microglial activation and disrupts hippocampal synaptic homeostasis, thereby contributing to cognitive impairment. Our findings suggest that microglial activation is associated with hippocampal synaptic degeneration and cognitive deficits following repeated propofol exposure. These results provide mechanistic insight into the neurobiological consequences of repeated propofol exposure in this experimental model and identify microglial activation as a potential therapeutic target for future investigation.

## 2. Results

### 2.1. Repeated Propofol Exposure Induces Robust Conditioned Place Preference and Cognitive Deficits

**Figure 1:**
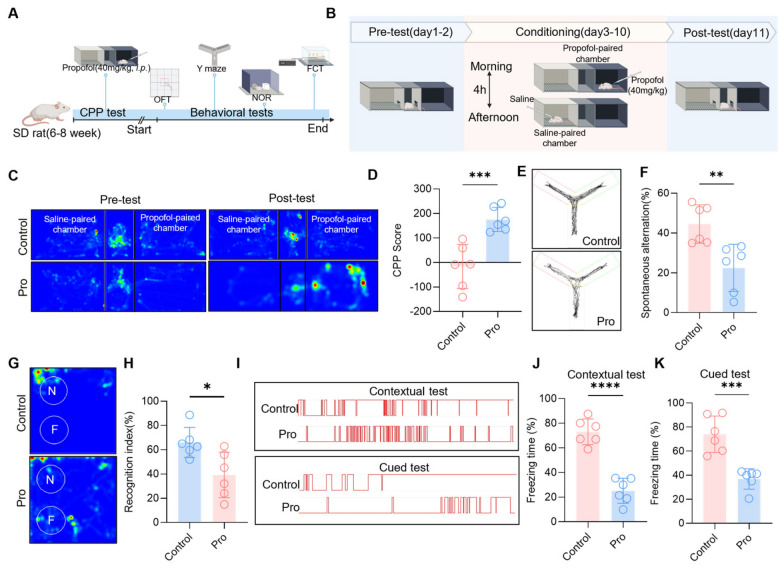
Repeated propofol exposure induces conditioned place preference and cognitive impairment. (**A**) Schematic of the experimental paradigm. (**B**) Schematic illustration of the CPP test. (**C**) Representative heat maps of the Control and Pro rats in the CPP test. (**D**) CPP scores in Control and Pro rats. (**E**) Representative diagram of Control and Pro rats in the Y-maze test. (**F**) The percentage of spontaneous alternation of the Control and Pro rats in the Y-maze test. (**G**) Representative heat maps of the Control and Pro rats in the NOR test. (**H**) The percentage of recognition index of the Control and Pro rats in the NOR test. (**I**) Representative diagram of the Control and Pro rats in the FCT. (**J**) The percentage of freezing time of the Control and Pro rats in the contextual test of the FCT. (**K**) The percentage of freezing time of the Control and Pro rats in the cued test of FCT. Data are presented as the mean ± SD. n = 6 for each group. Each dot represents an individual animal; pink and blue represent the Control and Pro groups, respectively. * *p* < 0.05; ** *p* < 0.01; *** *p* < 0.001; **** *p* < 0.0001. CPP, conditioned place preference; OFT, open field test; NOR, novel object recognition; FCT, fear conditioning test.

Following repeated propofol exposure, rats exhibited significantly increased CPP scores, indicating the establishment of propofol-associated reward memory (Figure 1C,D). No differences were observed in total distance traveled or mean velocity in the open field test, suggesting intact locomotor activity (Supplementary Figure S1A–C). To evaluate cognitive function, we performed Y-maze, novel object recognition (NOR), and fear conditioning tests. Propofol-treated rats displayed reduced spontaneous alternation in the Y-maze without changes in total arm entries, indicating impaired spatial working memory (Figure 1E,F and Figure S1D). In the NOR task, propofol-treated rats exhibited a lower recognition index than controls, reflecting impaired recognition memory (Figure 1G,H). Furthermore, freezing responses during both contextual and cued recall were significantly reduced in the fear conditioning test (Figure 1I–K). Together, these findings demonstrate that repeated propofol exposure induces conditioned place preference, indicative of addiction-like reward behavior, accompanied by significant impairments in learning and memory.

### 2.2. Transcriptomic Profiling Identifies Synapse-Related Gene Signatures in the Hippocampus Following Repeated Propofol Exposure

**Figure 2:**
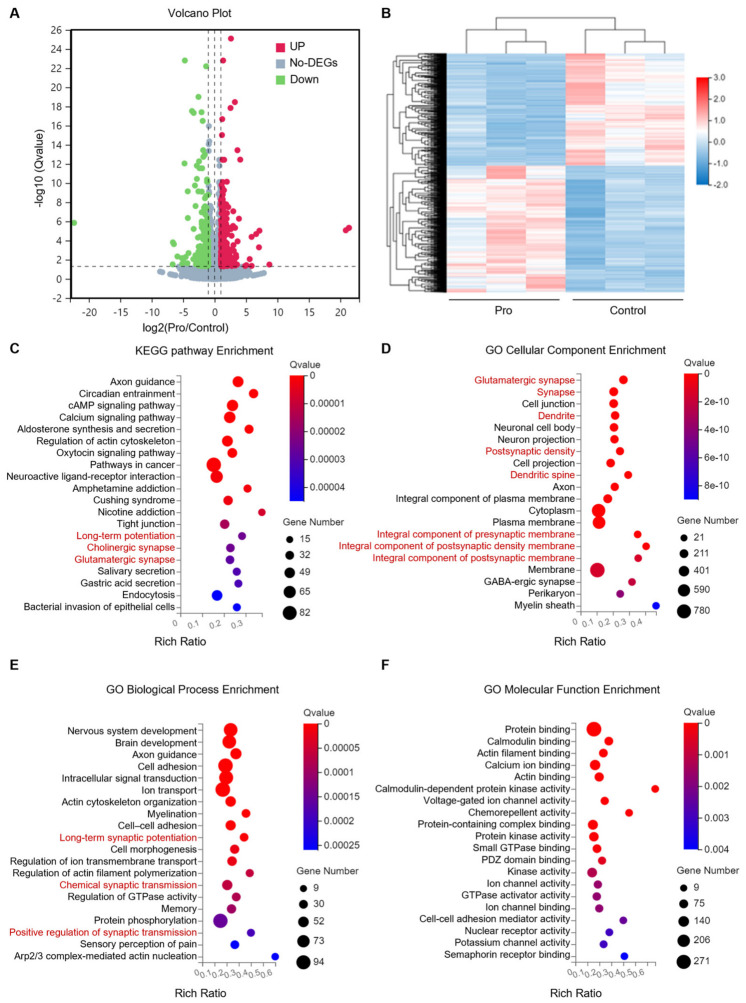
Transcriptomic profiling of the hippocampus in rats with repeated propofol exposure. (**A**) Volcano plot of differentially expressed genes in the hippocampus of rats in the Control and the Pro groups. (**B**) Heatmap of differentially expressed genes in the hippocampus of rats in the Control and the Pro groups. (**C**) KEGG pathway enrichment bubble chart. (**D**) GO cellular component enrichment bubble chart. (**E**) GO biological process enrichment bubble chart. (**F**) GO molecular function enrichment bubble chart. Synapse-related pathways and GO terms of particular relevance to the observed transcriptomic alterations are highlighted in red. n = 3 in each group. KEGG, Kyoto Encyclopedia of Genes and Genomes; GO, Gene Ontology.

RNA sequencing identified 1731 differentially expressed genes (DEGs) in the hippocampus of propofol-treated rats (defined as |log_2_ fold change| ≥ 1 and Q value < 0.05), including 918 upregulated and 813 downregulated genes (Figure 2A,B). Functional enrichment analyses showed that these DEGs were significantly enriched in synapse-related pathways and biological processes. Kyoto Encyclopedia of Genes and Genomes (KEGG) analysis identified significant enrichment of pathways such as long-term potentiation, cholinergic synapse, and glutamatergic synapse (Figure 2C). Gene Ontology (GO) enrichment analysis further showed that the DEGs were significantly enriched in synapse-related cellular components and biological processes, including synapses, dendrites, dendritic spines, postsynaptic compartments, and synaptic signaling (Figure 2D–F). Collectively, these transcriptomic findings suggest that repeated propofol exposure is associated with alterations in synapse-related transcriptional programs and identify candidate biological processes for subsequent experimental investigation.

### 2.3. Repeated Propofol Exposure Disrupts Hippocampal Synaptic Integrity

**Figure 3:**
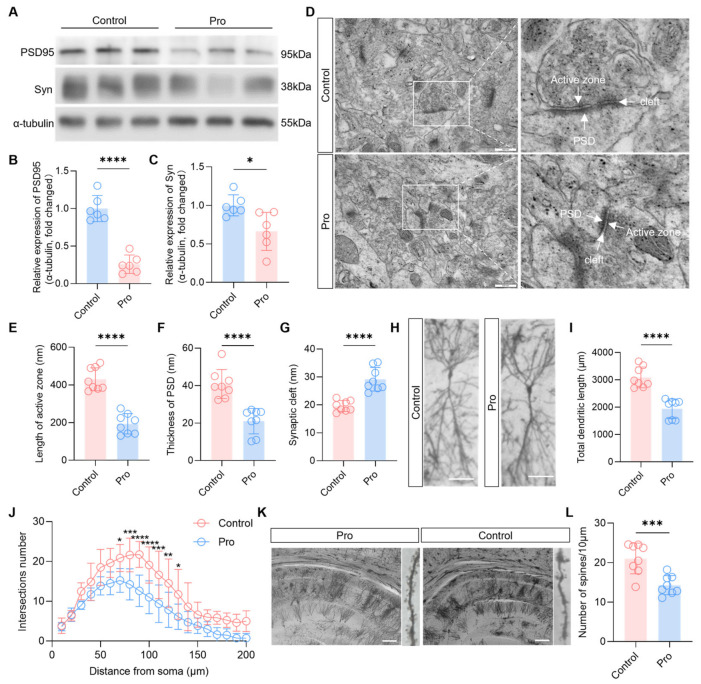
Repeated propofol exposure disrupts hippocampal synaptic integrity. (**A**) Representative Western blot images of PSD95 and Syn in the hippocampus from the Control and Pro rats. (**B**,**C**) Quantitative Western blot analysis of PSD95 and Syn in the hippocampus from the Control and Pro rats. (**D**) Representative transmission electron microscopy images of synapses in the hippocampus from the Control and Pro rats; scale bar = 500 nm. (**E**) The length of the synaptic active zone in the hippocampus of the Pro and Control rats. (**F**) The thickness of the PSD in the hippocampus of the Pro and Control rats. (**G**) The width of the synaptic cleft in the hippocampus of the Pro and Control rats. (**H**) Representative images of Golgi staining in the hippocampal CA1 region of the Pro and Control rats; scale bar = 50 μm. (**I**) Quantitative analysis of total dendritic length in the hippocampal CA1 region of the Pro and Control rats. (**J**) The Sholl analysis of hippocampal neuron morphology of the Pro and Control rats. (**K**) Representative images of dendritic spines in the hippocampal CA1 region of the Pro and Control rats; scale bar = 10 μm. (**L**) Quantification of dendritic spine density in the hippocampal CA1 region of the Pro and Control rats. The data are expressed as the mean ± SD. n = 6–8 per group. * *p* < 0.05; ** *p* < 0.01; *** *p* < 0.001; **** *p* < 0.0001. PSD95, postsynaptic density protein 95; Syn, synaptophysin.

To validate the synaptic alterations suggested by transcriptomic analysis, we examined hippocampal synaptic structure and plasticity. Propofol-treated rats exhibited significantly reduced expression of the synaptic proteins PSD95 and synaptophysin (Syn) (Figure 3A–C). Ultrastructural analysis further revealed shortened active zones, reduced postsynaptic density thickness, and widened synaptic clefts (Figure 3D–G). Consistent with these findings, Golgi staining demonstrated reduced dendritic length, decreased dendritic complexity, and lower dendritic spine density in hippocampal CA1 neurons following propofol exposure (Figure 3H–L). These results indicate that repeated propofol exposure induces profound synaptic and structural abnormalities in the hippocampus.

### 2.4. Repeated Propofol Exposure Induces Microglial Activation in the Hippocampus

**Figure 4:**
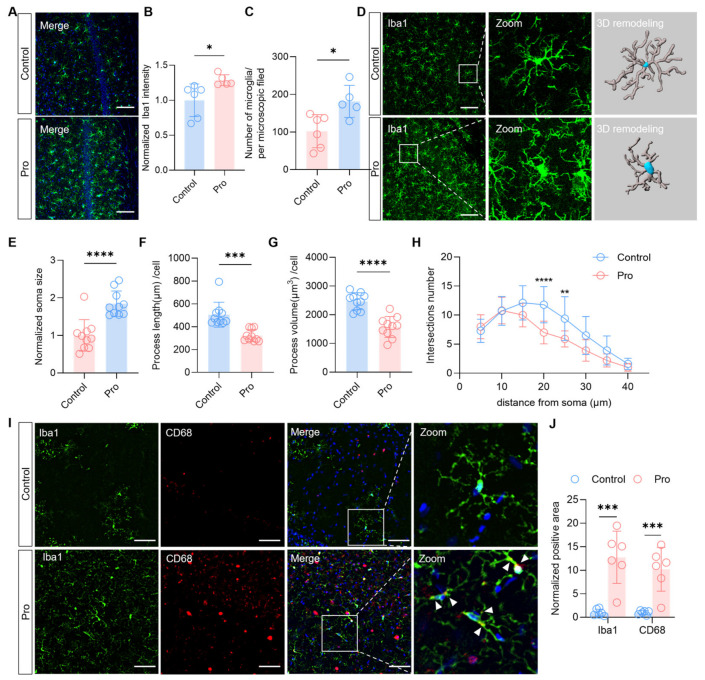
Morphological and phenotypic characterization of hippocampal microglia following repeated propofol exposure. (**A**) Representative images of immunofluorescence for Iba1 (green) and DAPI (blue) in the hippocampal CA1 region of Pro and Control rats; scale bar = 50 μm. (**B**) Quantitative analyses of Iba1 intensity in the hippocampal CA1 region of Pro and Control rats. (**C**) Quantitative analyses of Iba1^+^ cell numbers in the hippocampal CA1 region of Pro and Control rats. (**D**) Representative images of Iba1 immunofluorescence (green) and 3D remodeling (gray) of microglia in the hippocampal CA1 region of Pro and Control rats; scale bar = 50 μm. (**E**) Quantitative analyses of soma size of Iba1^+^ microglia in the hippocampal CA1 region of Pro and Control rats. (**F**) Quantitative analyses of the total process length of microglia in the hippocampal CA1 region of Pro and Control rats. (**G**) Quantitative analyses of the total process volume of microglia in the hippocampal CA1 region of Pro and Control rats. (**H**) The Sholl analysis of microglial morphology in the hippocampal CA1 region of Pro and Control rats. (**I**) Representative images of immunofluorescence for Iba1 (green), CD68 (red), and DAPI (blue) in the hippocampal CA1 region of Pro and Control rats; scale bar = 50 μm. (**J**) Quantitative analyses of immunofluorescence for Iba1 (green) and CD68 (red) in the hippocampal CA1 region of Pro and Control rats. The data are expressed as the mean ± SD. n = 6–10 per group. * *p* < 0.05; ** *p* < 0.01; *** *p* < 0.001; **** *p* < 0.0001. SD, standard deviation.

Iba1 immunostaining revealed a significant increase in both Iba1 fluorescence intensity and microglial density in the hippocampal CA1 region following repeated propofol exposure (Figure 4A–C). Three-dimensional reconstruction further demonstrated pronounced morphological alterations characteristic of microglial activation, including enlarged soma size, reduced process length, and decreased process volume (Figure 4D–G). Consistently, the Sholl analysis revealed a significant reduction in microglial process complexity in propofol-treated rats (Figure 4H). In addition, CD68 expression was markedly increased in hippocampal microglia following propofol exposure (Figure 4I,J), suggesting an activated microglial phenotype with increased lysosomal activity. Taken together, these findings demonstrate robust microglial activation in the hippocampus following repeated propofol exposure.

### 2.5. Minocycline Attenuates Repeated Propofol Exposure-Induced Microglial, Cognitive, and Synaptic Abnormalities

**Figure 5:**
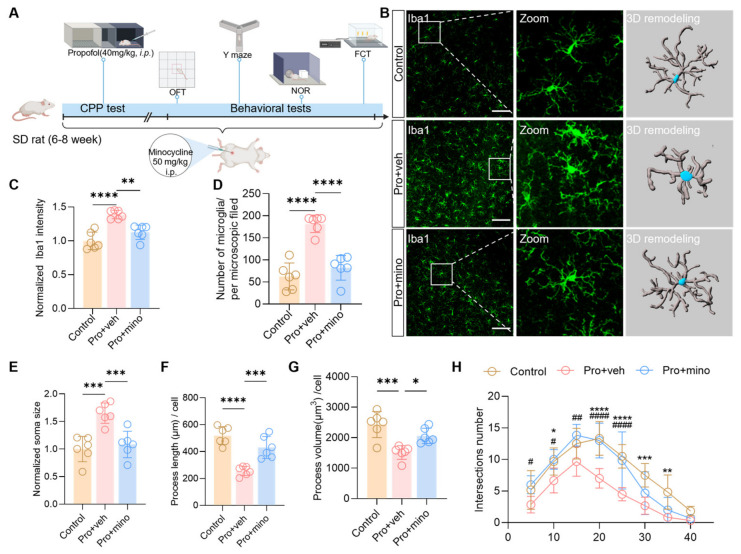
Effects of minocycline on microglial activation in the hippocampal CA1 region. (**A**) Schematic of the experimental paradigm. (**B**) Representative images of Iba1 immunofluorescence (green) and 3D remodeling (gray) of microglia in the hippocampal CA1 region of Control, Pro + veh, and Pro + mino rats; scale bar = 50 μm. (**C**) Quantitative analyses of Iba1 intensity in the hippocampal CA1 region of Control, Pro + veh, and Pro + mino rats. (**D**) Quantitative analyses of Iba1^+^ cell numbers in the hippocampal CA1 region of Control, Pro + veh, and Pro + mino rats. (**E**) Quantitative analyses of soma size of Iba1^+^ microglia in the hippocampal CA1 region of Control, Pro + veh, and Pro + mino rats. (**F**) Quantitative analyses of total process length of microglia in the hippocampal CA1 region of Control, Pro + veh, and Pro + mino rats. (**G**) Quantitative analyses of the total process volume of microglia in the hippocampal CA1 region of Control, Pro + veh, and Pro + mino rats. (**H**) The Sholl analysis of microglial morphology in the hippocampal CA1 region of Control, Pro + veh, and Pro + mino rats. *: Control vs. Pro + veh; #: Pro + veh vs. Pro + mino. The data are expressed as the mean ± SD. n = 6 per group. * *p* < 0.05; ** *p* < 0.01; *** *p* < 0.001; **** *p* < 0.0001; ^#^ *p* < 0.05; ^##^ *p* < 0.01; ^####^ *p* < 0.0001.

**Figure 6:**
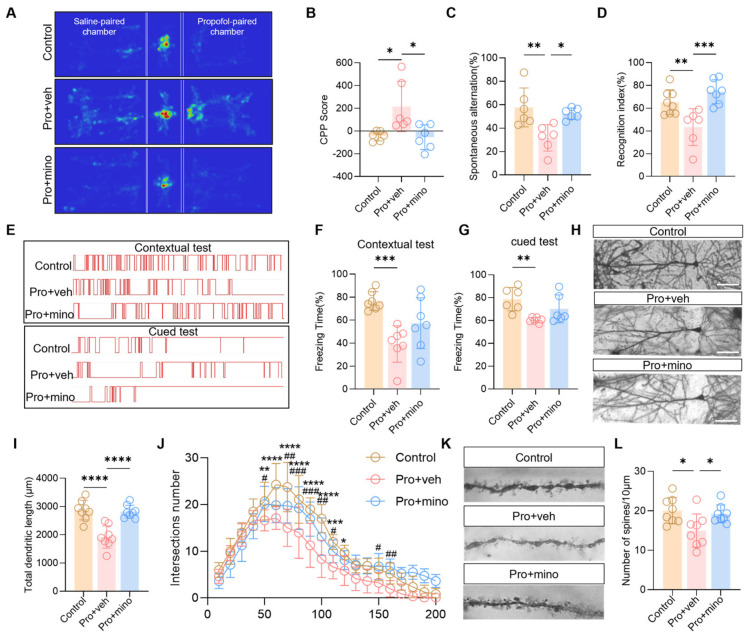
Effects of minocycline on behavioral and synaptic alterations induced by repeated propofol exposure. (**A**) Representative heat maps of the Control, Pro + veh, and Pro + mino rats in the CPP test. (**B**) The CPP scores of the Control, Pro + veh, and Pro + mino rats in the CPP test. (**C**) The percentage of spontaneous alternation of the Control, Pro + veh, and Pro + mino rats in the Y-maze test. (**D**) The percentage of recognition index of the Control, Pro + veh, and Pro + mino rats in the NOR test. (**E**) Representative diagram of the Control, Pro + veh, and Pro + mino rats in the FCT. (**F**) The percentage of freezing time of the Control, Pro + veh, and Pro + mino rats in the contextual test of FCT. (**G**) The percentage of freezing time of the Control, Pro + veh, and Pro + mino rats in the cued test of FCT. (**H**) Representative images of Golgi staining in the hippocampal CA1 region of the Control, Pro + veh, and Pro + mino rats; scale bar = 50 μm. (**I**) Quantitative analysis of total dendritic length in the hippocampal CA1 region of Control, Pro + veh, and Pro + mino rats. (**J**) The Sholl analysis of hippocampal neuron morphology of Control, Pro + veh, and Pro + mino rats. *: Control vs. Pro + veh; ^#^: Pro + veh vs. Pro + mino. (**K**) Representative images of dendritic spines in the hippocampal CA1 region of Control, Pro + veh, and Pro + mino rats. (**L**) Quantitative analysis of dendritic spine density in the hippocampal CA1 region of Control, Pro + veh, and Pro + mino rats. The data are expressed as the mean ± SD. n = 6 for each group. * *p* < 0.05; ** *p* < 0.01; *** *p* < 0.001; **** *p* < 0.0001. ^#^ *p* < 0.05; ^##^ *p* < 0.01; ^###^ *p* < 0.001.

To determine whether microglial activation contributes to propofol-induced hippocampal dysfunction, rats received minocycline throughout the conditioning period (Figure 5A). Minocycline treatment significantly reduced Iba1 immunoreactivity and microglial density in the hippocampal CA1 region and reversed the morphological features of microglial activation, including soma enlargement, process retraction, and reduced arbor complexity (Figure 5B–H). Functionally, minocycline significantly attenuated propofol-induced CPP without affecting locomotor activity (Figure 6A,B and Figure S2). Moreover, minocycline improved spatial working memory and recognition memory, as evidenced by increased spontaneous alternation in the Y-maze and an elevated recognition index in the novel object recognition task (Figure 6C,D). Minocycline treatment showed a nonsignificant trend toward increased freezing behavior (Figure 6E–G). Consistent with these behavioral improvements, minocycline markedly ameliorated abnormalities in hippocampal neuronal morphology, including dendritic length, dendritic complexity, and dendritic spine density (Figure 6H–L). Therefore, these findings support a role for microglial activation in propofol-induced cognitive and synaptic deficits, whereas pharmacological suppression of microglial activation alleviates these deficits.

## 3. Discussion

The addictive properties of propofol have been increasingly recognized; however, the consequences of repeated propofol exposure on cognitive function remain poorly understood. In the present study, repeated propofol exposure induced robust conditioned place preference, indicative of addiction-like reward behavior, accompanied by deficits in multiple domains of hippocampus-dependent memory. At the structural level, cognitive impairment was associated with widespread synaptic abnormalities, including reductions in synaptic proteins, dendritic spine loss, and ultrastructural synaptic disruption. Importantly, these alterations occurred in parallel with marked microglial activation, whereas pharmacological inhibition of microglial activation substantially ameliorated both synaptic pathology and cognitive dysfunction. Together, these findings demonstrate that repeated propofol exposure is associated with cognitive deficits, synaptic degeneration, and microglial activation. These observations further suggest that disruption of microglia-associated synaptic homeostasis may contribute to repeated propofol exposure-related cognitive impairment.

Previous investigations have primarily focused on the rewarding effects of repeated propofol exposure and their underlying neural mechanisms [36,37,38], whereas relatively little attention has been devoted to the associated cognitive consequences. Consistent with previous reports demonstrating the rewarding properties of propofol, repeated propofol exposure induced robust conditioned place preference, indicative of addiction-like rewarding effects. It should be noted, however, that the CPP paradigm primarily assesses conditioned rewarding effects and reward-associated memory rather than the full spectrum of addictive behaviors. Therefore, these behavioral findings should be interpreted within the scope of this experimental model. In addition to these reward-related behavioral effects, repeated propofol exposure also impaired multiple domains of hippocampus-dependent memory, including spatial working memory, recognition memory, and fear-associated learning. Notably, these cognitive deficits occurred in the absence of detectable changes in locomotor activity, suggesting that they were unlikely to be secondary to gross motor impairment. Although this finding does not fully exclude potential influences of motivational or anxiety-related factors, the overall behavioral profile is consistent with impairment of hippocampus-dependent cognitive function following repeated propofol exposure. Collectively, these findings extend current understanding of the neurobiological consequences of repeated propofol exposure beyond reward circuitry and identify hippocampal dysfunction as an important consequence of repeated propofol exposure.

A major finding of the present study is that repeated propofol exposure was associated with marked impairment of hippocampal synaptic integrity. Accumulating evidence suggests that maladaptive synaptic plasticity is a potential pathological substrate underlying addiction-associated cognitive dysfunction. Consistent with findings reported in cocaine, opioid, and alcohol addiction [39], transcriptomic profiling revealed enrichment of pathways associated with glutamatergic signaling and synaptic plasticity. Given the limited sample size of the RNA-seq analysis (n = 3 per group) and the absence of RT-qPCR validation of individual DEGs, these findings should be considered exploratory and hypothesis-generating and should be interpreted as identifying candidate biological processes for future investigation rather than as providing independent mechanistic evidence. Accordingly, the enrichment results were used to inform subsequent molecular and structural assessments. Western blotting demonstrated reduced synaptic protein expression, whereas transmission electron microscopy and Golgi staining revealed ultrastructural synaptic abnormalities and dendritic spine loss, respectively. These complementary findings provide convergent support for the broader synaptic phenotype suggested by the transcriptomic analysis. Together, these findings suggest that disruption of synaptic homeostasis may contribute to cognitive impairment following repeated propofol exposure.

An important finding of the present study is the association between microglial activation and hippocampal synaptic abnormalities following repeated propofol exposure. Increasing evidence suggests that aberrant microglial responses have been implicated in cognitive dysfunction [31,40,41]. Consistent with this concept, repeated propofol exposure induced robust microglial activation in parallel with widespread synaptic degeneration. Although direct evidence of microglia-mediated synaptic engulfment was not obtained, our findings demonstrate a close association among microglial activation, synaptic degeneration, and cognitive impairment following repeated propofol exposure. These observations raise the possibility that dysregulated microglia–synapse interactions may contribute to hippocampal pathology. However, this proposed mechanism remains speculative and requires direct experimental validation.

Pharmacological intervention further supported the involvement of microglial activation in propofol-induced neurobehavioral abnormalities. Inhibition of microglial activation with minocycline [42,43] markedly ameliorated synaptic degeneration and cognitive deficits, while also attenuating conditioned place preference, suggesting that microglial activation may be associated with addiction-related neuroplasticity and cognitive dysfunction. These findings are consistent with accumulating evidence that neuroimmune signaling contributes to reward processing, motivational behaviors, and synaptic plasticity. Although minocycline is widely used as a pharmacological inhibitor of microglial activation, it is not microglia-specific and may exert broader anti-inflammatory and neuroprotective effects. Therefore, the present findings support an association between microglial activation and the observed pathological changes rather than establishing a definitive causal role for microglia. Notably, our previous studies demonstrated that repeated propofol exposure activates TLR3-dependent neuroinflammatory signaling and promotes hippocampal neuronal senescence [22]. Considered together, these findings suggest that repeated propofol exposure may engage neuroimmune signaling, which could contribute to maladaptive microglial responses and synaptic abnormalities. Such alterations may compromise hippocampal network integrity and contribute to cognitive dysfunction. However, this proposed framework remains hypothetical, and the specific contribution of microglia-mediated synaptic remodeling requires direct experimental validation.

Several limitations should be considered. Cognitive function was assessed only during the repeated-exposure phase, precluding conclusions regarding the persistence of these deficits following propofol withdrawal. Although minocycline attenuated multiple pathological and behavioral abnormalities, it is not a microglia-specific intervention and may exert broader anti-inflammatory and neuroprotective effects. Therefore, the present pharmacological findings are most appropriately interpreted as supporting the involvement of an inflammation-associated component rather than establishing a definitive causal role for microglia. In addition, direct evidence of microglial engulfment of synaptic elements was not obtained. Accordingly, although our findings support an association between microglial activation and synaptic degeneration, whether aberrant microglia-mediated synaptic pruning contributes to these pathological changes remains a hypothesis requiring direct experimental validation. Furthermore, only adult male rats were included in this study; therefore, potential sex differences were not evaluated, and the present findings should be interpreted within the context of a male-rat model of repeated propofol exposure. Future studies combining microglia-specific genetic approaches, high-resolution imaging, direct assessment of microglia–synapse interactions, and both male and female animals will be required to establish causality, define the underlying molecular mechanisms, and determine the generalizability of these findings across sexes.

## 4. Materials and Methods

### 4.1. Animals

Adult male, 6–8-week-old Sprague–Dawley (SD) rats were purchased from the Dossy Life Science Company (Chengdu, China). Animal experiments were performed according to procedures approved by the Animal Care and Use Committee of Sichuan University (Permit Number: NO. 20220512001) and followed the Guide for the Care and Use of Laboratory Animals published by the US National Institutes of Health (NIH Publication No. 85-23, revised 1996).

Rats were randomly assigned to experimental groups before treatment. Investigators responsible for behavioral testing, image acquisition, Western blot quantification, histological analyses, transmission electron microscopy, Golgi staining, microglial three-dimensional reconstruction, and quantitative data analysis were blinded to group allocation throughout data acquisition and analysis.

### 4.2. Conditioned Place Preference

The CPP apparatus comprised two chambers (25 cm × 25 cm × 34 cm) connected by a small hallway (10 cm × 10 cm × 34 cm). The left and right chambers had different tactile and visual cues. In particular, the left chamber had a smooth floor and black wall, while the right chamber was distinguished by a grid floor and striped wall. Rats were placed in the CPP apparatus for acclimation and were allowed to explore freely for 15 min for 2 days before the CPP session. A standard CPP protocol was applied, including pre-test, conditioning, and post-test phases. During the pre-test phase, rats were released from the middle chamber of the CPP apparatus and allowed to explore the CPP apparatus for 15 min freely. The time spent in each chamber was recorded using the Smart 3.0 video tracking system (Reward, CA, USA) to evaluate the rat’s initial preference for the two chambers. The non-preferred chamber was chosen as the propofol-paired chamber, and the other chamber was designated as the saline-paired chamber.

The conditioning phase included seven successive days, with conditioning divided into two periods, morning and afternoon, each day, with a 4 h interval between the two periods. During the conditioning phase, rats were randomly assigned to the Control and Pro groups and received an injection of saline or propofol (1%, 10 mg/mL; AstraZeneca SpA, London, UK) (40 mg/kg, i.p.). The injection volume was 1 mL per 250 g body weight for both propofol and saline administration. The propofol dose was selected based on previous studies showing that repeated administration of this subanesthetic dose reliably induces reward-related behaviors without producing prolonged anesthesia, making it suitable for modeling repeated propofol exposure and abuse-like behavior in rodents [11]. Consistent with previous studies of repeated propofol exposure, saline was used as the control treatment [11,44]. Intraperitoneal administration was used because repeated intravenous injections are technically challenging in rats, and this route has been widely adopted in previous preclinical studies of repeated propofol administration to achieve consistent systemic drug exposure [11,44]. In the morning, propofol-treated rats received propofol and were immediately placed in the propofol-paired chamber for 30 min. In the afternoon, the rats received a saline injection and were placed into the opposite chamber for 30 min for seven consecutive days. Control rats received intraperitoneal injections of an equivalent volume of saline during both morning and afternoon conditioning sessions. During the post-test phase, the rats were placed in the central compartment and allowed to explore for 15 min freely. The time spent in the two main chambers was recorded. The CPP scores were calculated by the time spent in the propofol-paired chamber in the post-test phase minus that in the pre-test phase (CPP score = Time_post-test_ − Time_pre-test_).

### 4.3. Behavioral Tests

Behavioral assessments were initiated on the day following completion of the CPP post-test. To minimize the potential influence of acute pharmacological effects, all behavioral tests, including the Y-maze, novel object recognition, and fear conditioning tests, were performed before the daily propofol administration. Propofol was administered after completion of behavioral testing each day to maintain the repeated propofol exposure paradigm throughout the behavioral testing period while minimizing potential confounding effects associated with abrupt discontinuation of propofol exposure, including possible withdrawal-related behavioral changes.

#### 4.3.1. Open Field Test

The open field test (OFT) was conducted in an opaque box (100 × 100 × 48 cm) to test the locomotor functions of rats. The rats were transported into the behavioral testing room to acclimate to the environment for at least 1 h. The rats were placed in the center of the box and allowed to explore freely for 5 min. The activities of rats were recorded and analyzed with a Smart 3.0 video tracking system (Reward, CA, USA). Total travel distance and mean speed were used to assess locomotor function.

#### 4.3.2. Y-Maze Test

Spatial working memory was assessed with a Y-maze apparatus consisting of three arms (50 × 16 × 32 cm) labeled as A, B, and C. The rats were transported into the behavioral testing room to acclimate to the environment for at least 1 h. Each rat was placed in a fixed arm and allowed to explore freely for 8 min. The total number of arm entries and the sequence of arm entries were tracked and analyzed with the Smart 3.0 video tracking system (Reward, CA, USA). Alternation was defined as continuous exploration of all three arms (ABC, BCA, or CAB, but not BAB, CAC, or CBC). The percentage of spontaneous alternations was calculated as follows: Spontaneous alternation (%) = [number of alternations/(total number of arm entries − 2)] × 100%.

#### 4.3.3. Novel Object Recognition Test

Rats were tested for novel object recognition (NOR) in an opaque box (60 × 60 × 60 cm). The rats were transported into the behavioral testing room to acclimate to the environment for at least 1 h. Then, rats were habituated to the test box for 5 min in the absence of objects. During the training session, the rats were placed into the chamber with two identical objects and allowed to explore for 5 min. During the test session, one of the objects was replaced by a novel object, which differed from the familiar object in shape and color. The rats were placed in the chamber for 5 min, and the interaction time with two objects was recorded and analyzed using the Smart 3.0 video tracking system (Reward, CA, USA). Interaction with an object includes licking, sniffing, and touching the object. The recognition index was calculated as the percentage of time spent interacting with the novel object out of the total time spent exploring both objects in the test session.

#### 4.3.4. Fear Conditioning Test

Fear memory was assessed using a fear conditioning test (FCT) system (SANS, Nanjing China). Rats were placed in FCT chambers for 5 min to adapt to the experimental environment. During the training phase, after 2 min of exploration in the chamber, the rats received two tone-shock pairings (70 dB, 30 s) and electric shocks (1 mA, 1 s) with 2 min intertrial interval. The chambers were cleaned with 75% ethanol solution between trials. On day 2 (contextual test), the rats were placed in the same chambers as during training and received neither the tone nor the foot shock for 7 min. During the cued test phase, the rats were placed in changed chambers (inserting a Plexiglas floor over the shock grid and replacing Plexiglas on the wall). After 2 min, the tone (70 dB) from the training phase was presented for 3 min. The trials were recorded with a camera and analyzed using the FCT system (SANS, Nanjing, China).

### 4.4. Brain Tissue Collection

After behavioral tests, rats were deeply anesthetized and transcardially perfused with pre-cooled phosphate-buffered saline (PBS), followed by 4% paraformaldehyde (PFA). The brain was carefully removed and fixed overnight in a 4% PFA solution. Then, 30% sucrose was used for dehydration. The whole brain tissue was used to make brain slices for subsequent immunofluorescence staining. In addition, the hippocampus samples were harvested for Western blotting and RNA sequencing..

### 4.5. RNA Sequencing

Hippocampal tissues were collected and submitted to BGI Genomics for transcriptomic sequencing (Project ID: F23A040003252_RATowtnT; BGI, Shenzhen, China). Total RNA was extracted from hippocampal tissues, and RNA quality was assessed using a Fragment Analyzer system. All samples met the quality requirements for sequencing, with RNA quality numbers (RQNs) ranging from 9.7 to 10.0 and 28S/18S ratios ranging from 1.7 to 2.0. RNA libraries were prepared using the BGI transcriptome sequencing workflow and sequenced in paired-end 150 bp mode. After quality filtering, an average of 44.2 million clean reads and 6.63 Gb of clean bases were obtained per sample, with an average genome-mapping rate of 92.46%. Clean reads were aligned to the Rattus norvegicus reference genome (GCF_000001895.5_Rnor_6.0) using HISAT and to the reference gene set using Bowtie2. Gene expression abundance was expressed as fragments per kilobase of transcript per million mapped reads (FPKM). Differential expression analysis, data visualization, GO functional enrichment analysis, and KEGG pathway enrichment analysis were performed using the Dr. Tom platform (BGI Genomics; https://biosys.bgi.com; accessed on 5 December 2023). DEGs were identified using the criteria of |log_2_ fold change| ≥ 1 and Q value < 0.05. GO and KEGG enrichment analyses used the platform’s default background gene set, and all other analyses were performed using default parameters unless otherwise specified.

### 4.6. Western Blot

All hippocampal samples were lysed in ice-cold radioimmunoprecipitation assay (RIPA) lysis buffer (Tsingke Biotech, Beijing, China) and centrifuged at 12,000 rpm for 15 min at 4 °C. The supernatant was collected and quantified using a BCA kit (Beyotime, Shanghai, China) following the manufacturer’s instructions. Equal amounts of total protein were separated by sodium dodecyl sulfate–polyacrylamide gel electrophoresis (SDS-PAGE) and transferred onto polyvinylidene fluoride (PVDF) membranes (Millipore, Burlington, MA, USA). After blocking with 5% milk at room temperature for 1 h, the membranes were incubated overnight with primary antibodies at 4 °C. The following antibodies were used: anti-synaptophysin (Syn; 1:1000, ET1606-56; HUABIO, Hangzhou, China), anti-PSD95 (1:1000; 20665-1-AP; Proteintech, Wuhan, China), and anti-α-tubulin (1:5000; 11224-1-AP; Proteintech, Wuhan, China). Then, the membranes were incubated with the horseradish peroxidase-conjugated secondary antibody (Proteintech, Wuhan, China) for 1 h at room temperature. Chemiluminescent signals were detected under identical exposure settings for all samples within each experiment. Protein bands were quantified using ImageJ software (version 1.54p; National Institutes of Health, Bethesda, MD, USA), and the expression levels of Syn and PSD95 were normalized to α-tubulin. Each group included n = 6 independent biological samples. All samples were quantified using identical analysis parameters.

### 4.7. Golgi Staining

Golgi staining was performed using the FD Rapid Golgi Stain Kit (FD NeuroTech, Columbia, MD, USA). Freshly dissected rat brains were immersed in Solutions A and B for 2 weeks at room temperature and subsequently transferred to Solution C for 72 h in the dark. Coronal sections (150 μm thick) were prepared using a vibratome. The sections were stained using Solutions D and E, dehydrated through a graded ethanol series, and cleared in xylene. For image acquisition, images were obtained from three randomly selected, non-overlapping fields within the hippocampal CA1 region of each animal using an Olympus microscope (Olympus, Tokyo, Japan). Within each selected field, all well-impregnated neurons exhibiting complete morphology and minimal overlap with neighboring neurons were included for quantitative analysis. Morphometric parameters, including dendritic length, the Sholl intersections, and dendritic spine density, were quantified using ImageJ software. Measurements obtained from multiple neurons and fields were averaged for each animal, and the individual animal was treated as the independent experimental and statistical unit.

### 4.8. Transmission Electron Microscopy

Hippocampal CA1 tissue was rapidly dissected into approximately 1 mm^3^ blocks and immediately immersed in 2.5% glutaraldehyde. After fixing with 1% osmium tetroxide for 1.5 h, the samples were rinsed three times with double-distilled water. The samples were dehydrated through a graded ethanol series. After embedding, ultrathin sections were prepared using an ultramicrotome (Leica, Wetzlar, Germany) and stained with uranyl acetate and lead citrate. For ultrastructural analysis, three randomly selected, non-overlapping fields within the hippocampal CA1 region were imaged from each animal using a JEM-1400FLASH transmission electron microscope (JEOL, Tokyo, Japan). Within each selected field, all synapses with intact and clearly identifiable ultrastructural morphology were included for quantitative analysis. Synaptic ultrastructural parameters were quantified using ImageJ software. Measurements obtained from multiple synapses and fields were averaged for each animal, and the individual animal was treated as the independent experimental and statistical unit.

### 4.9. Immunofluorescence Staining

The brain tissues were cut into 40 μm thick sections using a freezing microtome (Leica, Wetzlar, Germany). The brain slices were rinsed three times with PBS and blocked for 1 h at room temperature. After blocking, the sections were incubated overnight at 4 °C with primary antibodies against ionized calcium-binding adaptor molecule 1 (1:1000; 019-19741; Iba1; Wako, Osaka, Japan) and CD68 (1:50; ab955; Abcam, Waltham, MA, USA). After rinsing three times with PBS, the slices were incubated with the corresponding secondary antibodies (Thermo Fisher Scientific, Waltham, MA, USA) in the dark for 1 h at room temperature. Then, images were obtained using fluorescence microscopy (Olympus, Tokyo, Japan) and laser microscopy (Leica, Wetzlar, Germany).

### 4.10. Three-Dimensional (3D) Reconstruction

Z-stack images were acquired along the *z*-axis using a Leica Stellaris confocal microscope. For each animal, three randomly selected, non-overlapping fields within the hippocampal CA1 region were analyzed. Microglial cells with clearly identifiable somata and processes were included for quantitative analysis. Three-dimensional reconstruction was performed using Imaris 10.1 software (Bitplane, Zurich, Switzerland). The Filament module was used to reconstruct microglial processes and quantify soma volume, total process length, total process volume, and process complexity. Measurements obtained from multiple microglial cells and fields were averaged for each animal, and the individual animal was treated as the independent experimental and statistical unit.

### 4.11. Minocycline Treatment

To investigate the involvement of microglial activation in cognitive impairment following repeated propofol exposure, the rats were intraperitoneally injected with saline or minocycline (Sigma-Aldrich, St. Louis, MO, USA) at a dose of 50 mg/kg/day starting from the first day of CPP conditioning and continuing once daily throughout the behavioral testing period until completion of the experimental protocol.

### 4.12. Statistical Analysis

All data are presented as the mean ± standard deviation (SD). Statistical analyses were conducted using GraphPad Prism 9. Data were assessed for normality using the Shapiro–Wilk test and for homogeneity of variance using the Brown–Forsythe test prior to statistical analysis. Comparisons between two groups were analyzed using an unpaired two-tailed Student’s *t*-test, whereas comparisons among multiple groups were analyzed by one-way analysis of variance (ANOVA) followed by Dunnett’s multiple comparisons test. *p* < 0.05 was considered statistically significant.

## 5. Conclusions

In conclusion, the present study provides evidence that repeated propofol exposure is associated with hippocampal synaptic pathology and cognitive impairment accompanied by robust microglial activation. Our findings support a close association between microglial responses, synaptic degeneration, and behavioral dysfunction in a rat model of repeated propofol exposure. These findings broaden our understanding of the neurobiological consequences of repeated propofol exposure and suggest that microglia-associated synaptic homeostasis may represent a potential therapeutic target for mitigating associated cognitive impairment. However, the therapeutic relevance of pharmacological modulation of microglial activity, including the use of minocycline, remains preliminary and requires further validation. Further studies are warranted to clarify the molecular mechanisms underlying microglia–synapse interactions following repeated propofol exposure.

## Data Availability

The original contributions presented in this study are included in the article/Supplementary Materials. Further inquiries can be directed to the corresponding author.

## Supplementary Materials

The following supporting information can be downloaded at https://www.mdpi.com/article/10.3390/ijms27146293/s1.

## Abbreviations

The following abbreviations are used in this manuscript:

CPP	Conditioned place preference
NOR	Novel object recognition
DEGs	Differentially expressed genes
KEGG	Kyoto Encyclopedia of Genes and Genomes
GO	Gene Ontology
PSD95	Postsynaptic density protein 95
Syn	Synaptophysin
SD	Sprague–Dawley
OFT	Open field test
FCT	Fear conditioning test
PBS	Phosphate-buffered saline
PFA	Paraformaldehyde
RIPA	Radioimmunoprecipitation assay
SDS-PAGE	Sodium dodecyl sulfate–polyacrylamide gel electrophoresis
PVDF	Polyvinylidene fluoride
