# Supplementary material for: Microglial Activation Is Associated with Hippocampal Synaptic Degeneration and Cognitive Deficits Following Repeated Propofol Exposure
Source: Int J Mol Sci. 2026 Jul 15;27(14):6293. doi: 10.3390/ijms27146293 (PMC13410312; doi:10.3390/ijms27146293)
Supplement: Supplementary file 1 [file ijms-27-06293-s001.zip › ijms-4403879-supplementary.pdf]

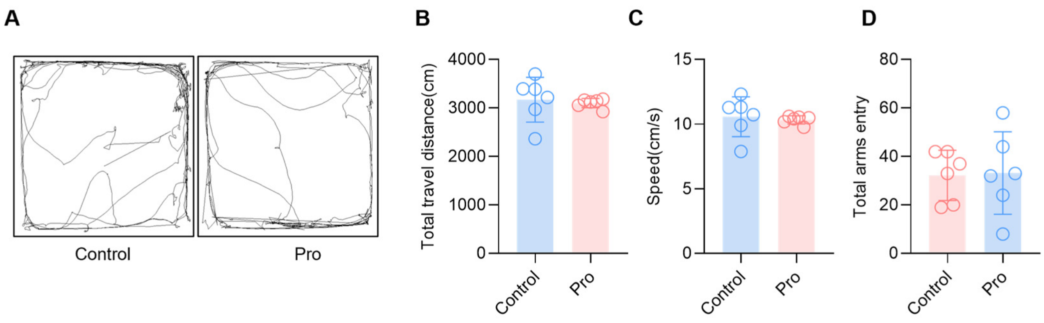

Supplementary Figure S1. Repeated propofol exposure does not affect locomotor activity. (A) The representative diagram of the Control and Pro rats in the OFT test. (B) Total distance traveled of the Control and Pro rats in the OFT test. (C) The mean speed of the Control and Pro rats in the OFT test. (D) The number of total arm entries of the Control and Pro rats in the Y-maze. The data are expressed as the mean ± SD. n=6 for each group. OFT, open-field test.

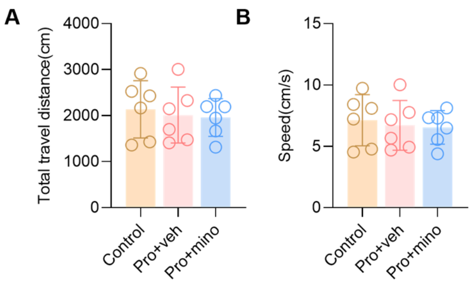

Supplementary Figure S2. Minocycline treatment did not impair the locomotor function of rats. (A) The total travel distance of the Control, Pro+veh, and Pro+mino rats in the OFT test. (B) The mean speed of the Control, Pro+veh, and Pro+mino rats in the OFT test. The data are expressed as the mean ± SD. n=6 for each group. OFT, open-field test.
